# Changes in Gene Expression Associated with FTO Overexpression in Mice

**DOI:** 10.1371/journal.pone.0097162

**Published:** 2014-05-19

**Authors:** Myrte Merkestein, James S. McTaggart, Sheena Lee, Holger B. Kramer, Fiona McMurray, Mathilde Lafond, Lily Boutens, Roger Cox, Frances M. Ashcroft

**Affiliations:** 1 Henry Wellcome Centre for Gene Function, Department of Physiology, Anatomy; and Genetics, University of Oxford, Parks Road, Oxford, United Kingdom; 2 Medical Research Council Harwell, Mammalian Genetics Unit, Harwell Science and Innovation Campus, Harwell, Oxford, United Kingdom; CRCHUM-Montreal Diabetes Research Center, Canada

## Abstract

Single nucleotide polymorphisms in the first intron of the fat-mass-and-obesity-related gene *FTO* are associated with increased body weight and adiposity. Increased expression of FTO is likely underlying this obesity phenotype, as mice with two additional copies of *Fto* (FTO-4 mice) exhibit increased adiposity and are hyperphagic. FTO is a demethylase of single stranded DNA and RNA, and one of its targets is the m6A modification in RNA, which might play a role in the regulation of gene expression. In this study, we aimed to examine the changes in gene expression that occur in FTO-4 mice in order to gain more insight into the underlying mechanisms by which FTO influences body weight and adiposity. Our results indicate an upregulation of anabolic pathways and a downregulation of catabolic pathways in FTO-4 mice. Interestingly, although genes involved in methylation were differentially regulated in skeletal muscle of FTO-4 mice, no effect of FTO overexpression on m6A methylation of total mRNA was detected.

## Introduction

In 2007, a genome-wide association study (GWAS) revealed an association between a single nucleotide polymorphism (SNP) in the first intron of the fat-mass-and-obesity-related gene *FTO* and increased BMI and adiposity.[1] Subjects carrying two copies of the risk allele were, on average, 3 kgs heavier and had a 1.7-fold increased risk for developing obesity.[1] Subsequently, this result has been replicated in various populations and across different age groups (reviewed in [2]). Increased expression of FTO probably underlies the obesity phenotype, as transcripts of the risk allele are produced more abundantly than the non-risk allele.[3], [4] Indeed, mice with a constitutive knock-out of *Fto* [5]–[7], or with a partial loss-of-function mutation in *Fto* [8], display a lean phenotype. Conversely, mice carrying one or two additional copies of *Fto* (FTO-3 and FTO-4 mice) exhibit increased body weight, fat mass and food intake.[9]

FTO is an Fe(II) and 2-oxoglutarate-dependent demethylase of single stranded DNA and RNA.[10] Its main targets appear to be 6-methyladenosine (m6A) (DNA/RNA), 3-methyluracil (m3U) (RNA) and 3-methylthymidine (m3T) (DNA/RNA).[10]-[12] Of special interest is the m6A modification, which is the main substrate of methylation in mRNA. This modification has been suggested to affect RNA processing [13]–[15], RNA transport [16]–[18] and translation efficiency [19]. m6A RNA methylation is catalysed by methyltransferases (METTL14 and METTL3) in association with the splicing factor WTAP.[20], [21] Silencing of any of these compounds resulted in decreased levels of m6A and increased abundance of target mRNA transcripts. [20] Furthermore, m6A is recognised by the ‘reader’ protein YTHDF2 which causes the mRNA to be localised to mRNA decay sites.[22] This indicates that reversible m6A modifications can influence the stability of mRNA and hence regulates its lifespan, with increased m6A methylation leading to a decrease in translation. Two studies have identified m6A-containing mRNAs in mouse brain [23] and mouse liver [24]. They reported an enrichment of m6A sites around the stop codon, which also suggests a role in translational control and that m6A methylation of mRNA plays a key role in the regulation of gene expression.

In summary, FTO is known to be involved in the regulation of body weight and adiposity, and is able to demethylate single stranded DNA and RNA at m6A, m3U and/or 3mT. How this function contributes to the physiological effects of FTO overexpression is still unknown. Alterations in gene expression and m6A methylation have been documented for ghrelin in response to FTO overexpression [4] and of genes involved in dopaminergic signalling in response to FTO knockout [25]. However, the full range of mRNAs affected by FTO is unknown.

In this study, we aimed to examine the changes in gene expression that occur in FTO-4 mice in order to gain more insight into the underlying mechanisms by which FTO influences body weight and adiposity. Our results indicate an upregulation of anabolic pathways and a downregulation of catabolic pathways in FTO-4 mice. Interestingly, although genes involved in methylation were differentially regulated in skeletal muscle of FTO-4 mice, no effect of FTO overexpression on m6A methylation levels of total mRNA was detected.

## Materials and Methods

### Animals

Mice expressing 2 additional copies (4 in total) of FTO on a C57BL/6J background (FTO-4) were generated as described previously. [9] Wild-type C57BL/6J littermates were used as controls. Genotyping was performed on DNA extracted by a DNeasy blood and tissue kit (Qiagen, USA). All experiments were carried out on 5–6 week old male mice maintained in a temperature and humidity controlled room on a 12∶12 light-dark cycle (lights on at 7am) with *ad libitum* access to water and food (SDS Rat and Mouse No. 3 Breeding diet (RM3) containing 11.5 kcal% fat, 23.93 kcal% protein and 61.57 kcal% carbohydrate). Mice were killed by cervical dislocation at 1pm. Abdominal white adipose tissue (WAT), skeletal muscle and brain were rapidly dissected and kept on dry ice and subsequently at −80°C until RNA or protein extraction.

### Ethics Statement

Experiments were conducted in accordance with the UK Animals (Scientific Procedures) Act (1986) and following approval by the University of Oxford local Ethics Review Committee. This study was performed under certificate of designation number 30/2306 and project license 30/2668 following approval by the University of Oxford Departments of Physiology, Anatomy and Genetics and Experimental Psychology Joint Departmental Ethics Review Committee.

### RNA extraction

Total ribonucleic acid (RNA) from abdominal white adipose tissue (WAT), skeletal gastrocnemius muscle, hypothalamus and cerebellum of wild-type (n = 4) and FTO-4 (n = 4) mice was extracted using an RNeasy Mini Kit (brain), RNeasy fibrous Mini Kit (skeletal muscle) or RNeasy Lipid Tissue Mini Kit (WAT) (Qiagen, USA) according to the manufacturer's protocol. The RNA concentration was measured in triplicate with a NanoDrop spectrophotometer (Thermo Scientific) and RNA integrity was assessed using an Agilent 2100 BioAnalyzer. Only samples with an RNA Integrity Number of ≥7.0 were used for microarray and RT-PCR studies.

### Microarray

Labelled sense ssDNA for hybridization was generated from 300 ng (cerebellum, hypothalamus, muscle) or 500 ng (WAT) starting RNA with the Ambion WT Expression Kit, the Affymetrix WT Terminal Labeling and Controls Kit and the Affymetrix Hybridization, Wash, and Stain Kit, according to the manufacturer's instructions. Sense ssDNA was fragmented and the distribution of fragment lengths measured on a BioAnalyser. The fragmented ssDNA was labeled and hybridized to the Affymetrix GeneChip Mouse Gene 1.0 ST Array (Affymetrix). Chips were processed on an Affymetrix GeneChip Fluidics Station 450 and Scanner 3000.

Microarray data was PLIER normalised independently for each tissue using GeneSpring GX11.0 (Agilent). Differentially expressed genes were identified using the Bioconductor limma package within GeneSpring. The R script used limma to fit a linear model and compute moderated t-statistics for all the genes; p-values were calculated using empirical computation from 10,000 permutations. Subsequently a Benjamini and Hochberg multiple testing correction was applied with a p value cut off of ≤0.05. All p values were <0.0001. Finally a 1.5 fold change difference between wild-type and FTO-4 was applied. GO-Elite was employed to assess significantly regulated gene ontology (GO) terms in the data sets described above. At least 3 genes and ≥10% genes in a GO term needed to be changing with a permuted P value of ≤0.05 to be included. Microarray data are available in the ArrayExpress database (www.ebi.ac.uk/arrayexpress) under accession number E-MTAB-2331.

### RT-qPCR experiments

For each sample, 1 µg of total RNA was reverse transcribed in a 20 µl final volume using the High Capacity complementary DNA (cDNA) Reverse Transcription kit (Applied Biosystems) according to the manufacturer's protocol. As a control, another 1 µg of total RNA was processed identically but without reverse transcriptase (non-RT control). cDNA samples were subsequently diluted to 4 ng/µl in nuclease-free water (Sigma) and stored at -80°C until further use.

Reverse transcription quantitative PCR (RT-qPCR) was carried out with an ABI Prism 7000 Sequence Detection System (Applied Biosystems). Reactions were performed in triplicate and contained 12.5 µl Power SYBR Green Master Mix, 300 nM forward and reverse primers, 20 ng of cDNA template (or non-RT control or nuclease-free water) and nuclease-free water to a total volume of 25 µl. The RT-qPCR reaction consisted of an initial denaturation for 10 min at 95°C, followed by 40 cycles of 95°C for 15 sec and 60°C for 60 seconds. Threshold cycle (Ct) values were calculated using ABI SDS 3000 software (Applied Biosystems).

Primers for targets to be validated (see Table S1 in File S1) and for three reference genes (HSPA8, HPRT1, ACTB) were designed with Geneious Pro software (Biomatters Inc) and NCBI sequence information. Primer specificity was confirmed by BLAST searches, the appearance of a single band on gel electrophoresis and melt curve analysis. Primer efficiencies were calculated as described previously [26] and used to calculate relative quantities of transcripts. Normalisation factors (the geometric mean [27]) were calculated with the geNorm applet for Microsoft Excel based on the transcript levels of the three reference genes. These were used to normalize the expression levels of the transcripts for differences in the amount of input material. Ct values for amplification of the transcripts of interest were significantly higher for non-RT controls (32.07±0.70) than for cDNA templates (22.39±0.68). The minimum difference in Ct values we observed between non-RT controls and cDNA samples was 5.63 cycles. Hence, contamination by genomic material was unlikely to influence the RT-qPCR data presented here.

To examine whether m6A might influence primer annealing and hence our microarray and qPCR results, PCR products with or without m6A were generated with 3 primer sets (HSPA8, HPRT1, ACTB). Equal amounts of these DNA templates were used to perform qPCR as described above.

### Cell culture

Mouse embryonic fibroblasts (MEFs) of wild-type and FTO-4 C57BL/6J mouse embryos were cultured at 37°C and 7% CO_2_ in Dulbecco's Modified Eagles Medium Glutamax (Gibco, Paisley, Scotland) supplemented with 10% (v/v) fetal calf serum, 100 units/ml penicillin, 100 units/ml streptomycin, 1x non-essential amino acids (Gibco, Paisley, Scotland) and 50 µM beta-mercaptoethanol (Gibco, Paisley, Scotland). Total RNA was isolated using an RNeasy Mini Kit (Qiagen, USA) according to the manufacturer's instructions. An aliquot of total RNA was taken for subsequent analysis. The remainder of the total RNA was used for mRNA extraction with the polyATtract system (Promega).

### Hydrolysis of RNA and isolation of nucleosides by solid phase extraction

RNA was incubated with 2U of nuclease P1 (Sigma) in a solution containing 25 mM NaCl and 2.5 mM of ZnCl_2_. After 1 hour incubation at 37°C, 10 U alkaline phosphatase (NEB) and NH_4_HCO_3_ (to an end concentration of 0.05 M) were added, followed by an additional 1 hour incubation at 37°C. Subsequently, 20 mM acetic acid was added to the reaction mixture to a total volume of 1 ml. A strong cation exchange column (Telos SCX 50 mg/1 ml) was equilibrated with 1 ml methanol and washed with 1 ml 20 mM acetic acid, after which the samples were loaded onto the colum Following a washing step with 1 ml 20 mM acetic acid, nucleosides were eluted with 1 ml methanol containing 2.8% ammonium hydroxide. Finally, samples were dried using a speed-vac, and the pellet was dissolved in buffer A containing 95% water, 5% acetonitrile and 2.5 mM ammonium.

### Liquid chromatography- mass spectrometry (LC-MS) for quantification of nucleosides

Samples were analysed by LC-MS on a Ultimate 3000 high performance liquid chromatography (HPLC) system (Dionex, Camberley, UK) coupled via the standard electrospray ionisation (ESI) interface to an Amazon ETD ion trap mass spectrometer (Bruker Daltonics) equipped with a nitrogen generator (Dominick Hunter, LCMS20-1). The HPLC system consisted of a solvent rack and degasser (SRD-3600), a dual gradient pump (DGP-3600M), a flow manager (FLM-3100) and a well plate autosampler (WPS-3000T). The mass spectrometer was controlled by TrapControl software version 7.0 and the LC-MS/MS system was controlled by HyStar software version 3.2.

RNA hydrolysates were separated by HPLC and a 1.0µl aliquot of the sample was injected per run. The chromatographic separation was carried out at a flow rate of 250µl/min on a Thermo Accucore HILIC column (2.1×100 mm, 2.6µm particle size, Hemel Hempstead, UK) at 35°C with isocratic elution using 95% acetonitrile, 5% water 2.5 mM ammonium formate, pH 3.8 as solvent. The mass spectrometer was operated in full scan MS mode (75 to 500 m/z scan range) and ionization was achieved at a capillary voltage of 4500 V, a nitrogen gas flow of 8 l/min and 15 psi pressure and a temperature of 300°C. The ion trap was set to an ion charge control (ICC) target of 250,000 and a maximum accumulation time of 200 ms. The instrument was calibrated at least once a month using an ESI-T tuning mix (Agilent) by performing scan, isolation and fragmentation calibrations (Bruker Daltonics).

### Data analysis

Analysis was performed using Quantanalysis software (version 2.0, Bruker Daltonics) and chromatograms of mass values for analytes (adenosine (A), N6-methyl adenosine (m6A); [M+H^+^] m/z 268.0 Da(A) and 282.0 Da(m6A)) and internal standard (^13^C5-adenosine (^13^C5-A), [M+H^+^] m/z 273.0 Da). A Gaussian smoothing algorithm (smoothing width 1s, 1 cycle) was applied to the chromatograms and automatic peak detection parameters for analyte and internal standard were: retention time (A, ^13^C5-A: 3.8 min, m6A: 2.9 min) and retention time window (0.3 min). Peak area ratios of nucleoside to internal standard (y axis) were plotted against the plasma concentrations (x-axis). A linear regression analysis of the calibration standards was carried out using the least squares method. Calibration, QC and study samples were analysed by triplicate injection on the LC-MS/MS system and the determined value was taken as the arithmetic mean of the three measurements.

## Results

Previous studies have shown that the body weights of wild-type (WT) mice and those overexpressing FTO (FTO-4 mice) start to diverge at 5 weeks of age. [9] We therefore used 5-week-old male mice for this study. We anticipate changes in gene expression underlying the FTO overexpression phenotype will be present at this age, but not changes that occur as a secondary consequence of increased body weight. We found no significant difference in body weight between 5-week-old FTO-4 and WT mice (FTO-4, 18.1±1.9 g; WT, 14.2±1.2 g; p = 0.13).

Overexpression of FTO in FTO-4 mice was confirmed by qPCR at 5 weeks of age. We found the highest expression of FTO in WAT (5.4-fold greater than WT), followed by cerebellum (2.1-fold), gastrocnemius muscle (1.5-fold) and hypothalamus (1.5-fold). Differences in the extent of overexpression between tissues of FTO-4 mice were also described previously, being greatest in muscle, but also significant in brain and white adipose tissue (WAT). [9]

### Influence of m6A on DNA amplification

**Figure 1:**
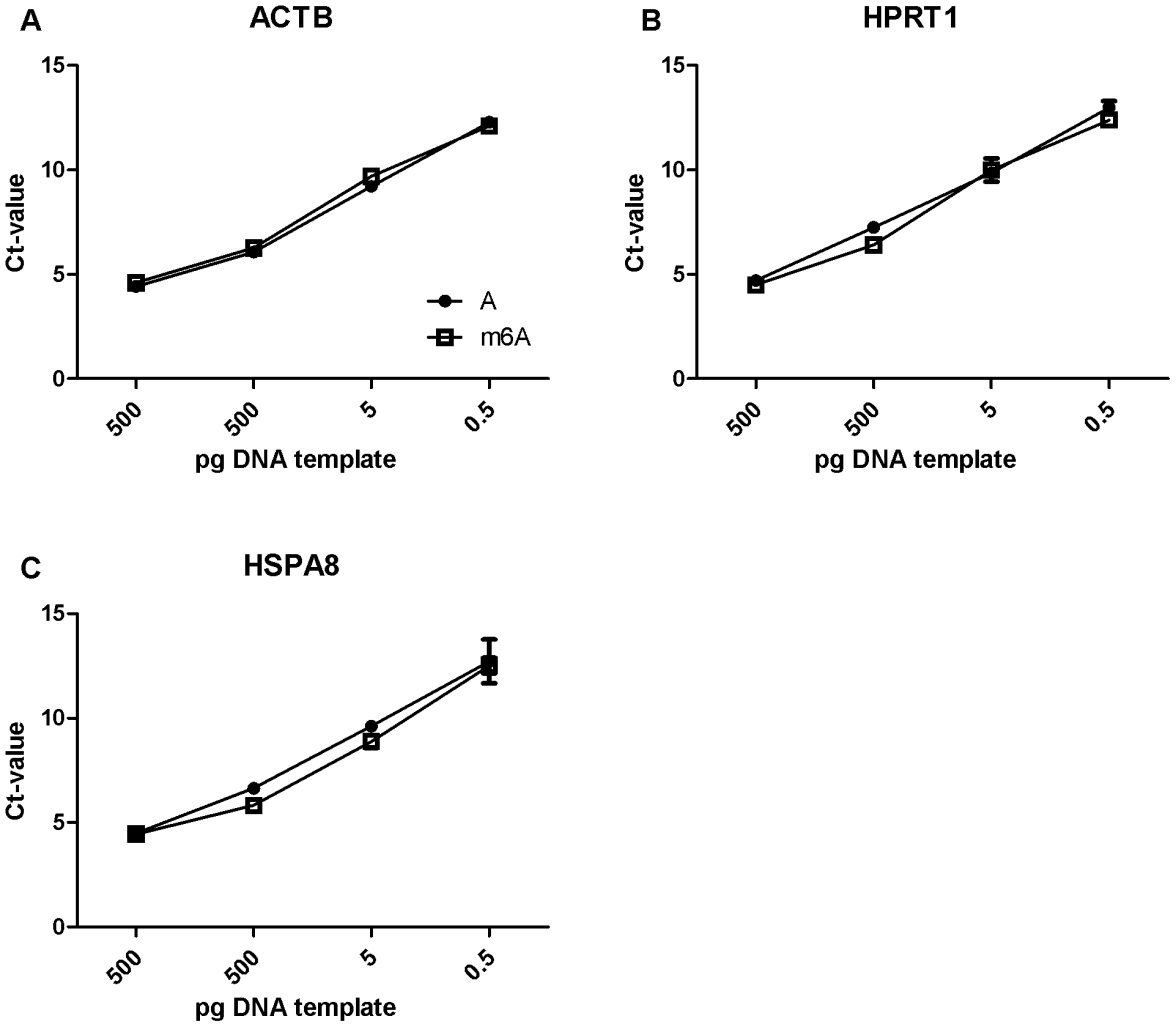
qPCR analysis of the effect of m6A on DNA amplification. qPCR analysis using different concentrations of the DNA templates ACTB (A), HPRT1 (B) and HSPA8 (C) containing m6A (open squares) or unmethylated adenosine (black circles) revealed that m6A does not interfere with DNA amplification.

Because it has been reported that FTO demethylates 6-methyladenosine (m6A) in mRNA [10], [12], we first examined whether m6A might affect primer annealing and subsequent amplification, and hence potentially influence both the qPCR and microarray analysis. We generated PCR products (ACTB, HPRT1, HSPA8) with and without m6A and used these as templates for qPCR analysis. As shown in Figure 1, no differences in threshold cycle (Ct) were observed with ACTB (A), HPRT1 (B) or HSPA8 (C) at any of the template concentrations used.

### Gene expression changes

**Figure 2:**
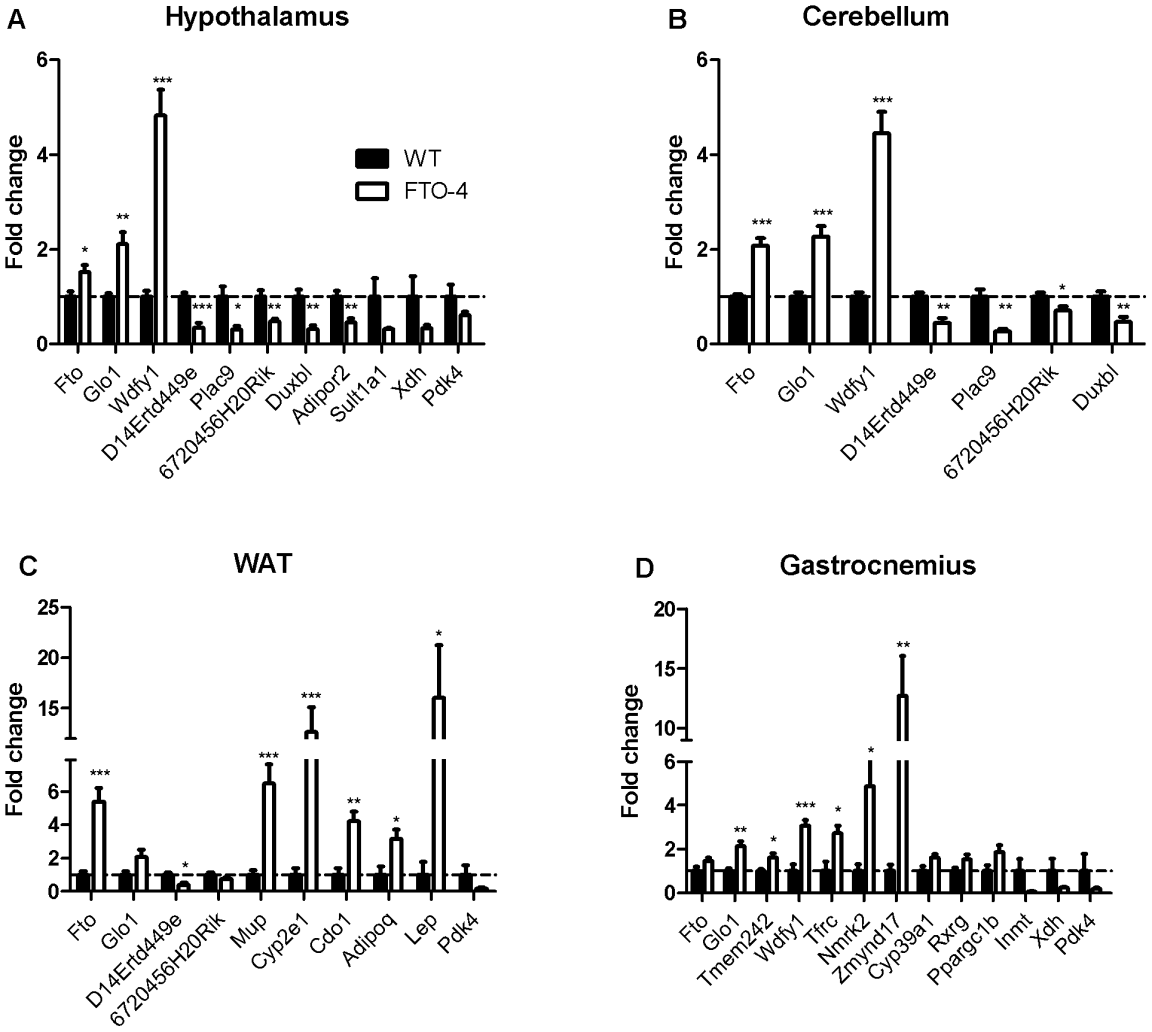
Differences in gene expression between WT and FTO-4 mice based on microarray data. Expression of the indicated genes in the cerebellum (A), hypothalamus (B), WAT (C) and gastrocnemius (D) of wildtype (WT, black bars) and FTO-4 (white bars) mice. Data are expressed as the fold change compared to wildtype. Significance was tested using Student's t-test to compare FTO-4 to WT. ***p<0.001, **p<0.01, *p<0.05.

Differences in gene expression between FTO-4 and WT mice were examined by microarray at 5 weeks of age in cerebellum, hypothalamus, gastrocnemius muscle and WAT. An overview of all differentially expressed genes per tissue can be found in File S2. Tables 1 and 2 list the top 10 genes that showed the greatest increase and decrease in expression per tissue. Table 3 lists all genes that showed altered expression in FTO-4 mice which have previously been associated with obesity. We selected several of these for validation by qPCR (Figure 2). We focused on genes with the highest fold-change, genes that showed altered expression in multiple tissues, and genes that have been previously implicated in obesity. All genes showed equivalent changes in both microarray and qPCR experiments (Table S2 in File S1).

**Table 1:** Top 10 genes that showed the highest amount of overexpression in FTO-4 mice per tissue.

Most upregulated genes			
Hypothalamus			
*Gene name*	*Gene symbol*	*Entrez ID*	*Fold change*
Glyoxalase 1	Glo1	109801	2.17
WD repeat and FYVE domain containing 1	Wdfy1	69368	1.91
Ribonuclease, RNase A family, 1 (pancreatic)	Rnase1	19752	1.91
Expressed sequence AI413582	AI413582	106672	1.67
Solute carrier family 38, member 5	Slc38a5	209837	1.67
Transmembrane protein 242	Tmem242	70544	1.65
Ermin, ERM-like protein	Ermn	77767	1.63
Carbonic anhydrase 2	Car2	12349	1.63
Epithelial membrane protein 1	Emp1	13730	1.59
Cholinergic receptor, nicotinic, alpha polypeptide 3	Chrna3	110834	1.57
Cerebellum			
WAP four-disulfide core domain 10	Wfdc10	629756	4.01
WD repeat and FYVE domain containing 1	Wdfy1	69368	1.99
Solute carrier family 38, member 5	Slc38a5	209837	1.84
Ribonuclease, RNase A family, 1 (pancreatic)	Rnase1	19752	1.82
Expressed sequence AI413582	AI413582	106672	1.78
Predicted gene 3435	Gm3435	100041621	1.73
Transmembrane protein 242	Tmem242	70544	1.66
Epithelial membrane protein 1	Emp1	13730	1.64
Predicted gene 13931	Gm13931	668825	1.62
Predicted gene 7168	Gm7168	635895	1.57
White adipose tissue			
Major urinary protein family	Mup	-	5.27
Eosinophil-associated, ribonuclease A family, member 11	Ear11	93726	4.88
Carbonic anhydrase 3	Car3	12350	4.20
Cytochrome P450, family 2, subfamily e, polypeptide 1	Cyp2e1	13106	3.83
UDP-Gal:betaGlcNAc beta 1,3-galactosyltransferase, polypeptide 2	B3galt2	26878	3.50
Adiponectin, C1Q and collagen domain containing	Adipoq	11450	3.26
Carboxylesterase 1D	Ces1d	104158	3.19
Regulator of G-protein signaling 13	Rgs13	246709	3.17
Galactosidase, beta 1-like 2	Glb1l2	244757	3.06
Myeloblastosis oncogene-like 1	Mybl1	17864	3.06
Gastrocnemius muscle			
Nicotinamide riboside kinase 2	Nmrk2	69564	13.93
Zinc finger, MYND domain containing 17	Zmynd17	74843	6.12
Cbp/p300-interacting transactivator, with Glu/Asp-rich carboxy-terminal Domain, 4	Cited4	56222	4.86
Transferrin receptor	Tfrc	22042	4.41
Potassium voltage-gated channel, subfamily G, member 4	Kcng4	66733	4.32
Dual specificity phosphatase 10	Dusp10	63953	3.44
Parkinson disease (autosomal recessive, juvenile) 2, parkin	Park2	50873	3.24
Integrin beta 6	Itgb6	16420	3.04
Inhibin alpha	Inha	16322	2.90
Ret proto-oncogene	Ret	19713	2.83

**Table 2:** Top 10 genes that showed the highest amount of downregulation in FTO-4 mice per tissue.

Most downregulated genes			
Hypothalamus			
*Gene name*	*Gene symbol*	*Entrez ID*	*Fold change*
Growth hormone	Gh	14599	−14.89
Cyclin-dependent kinase inhibitor 1A (P21)	Cdkn1a	12575	−2.19
Cyclin-dependent kinase inhibitor 1B	Cdkn1b	12576	−2.00
Thioredoxin interacting protein	Txnip	56338	−1.92
Zinc finger and BTB domain containing 16	Zbtb16	235320	−1.91
DNA segment, Chr 14, ERATO Doi 449, expressed	D14Ertd449e	66039	−1.8
Sulfotransferase family 1A, phenol-preferring, member 1	Sult1a1	20887	−1.79
RIKEN cDNA 6720456H20 gene	6720456H20Rik	218989	−1.78
Serum/glucocorticoid regulated kinase 1	Sgk1	20393	−1.74
FK506 binding protein 5	Fkbp5	14229	−1.68
Cerebellum			
Zinc finger and BTB domain containing 16	Zbtb16	235320	−2.44
Nuclear receptor interacting protein 2	Nrip2	60345	−2.28
Glutathione peroxidase 3	Gpx3	14778	−2.23
Prolactin family 2, subfamily c, member 3	Prl2c3	18812	−2.14
Alanine-glyoxylate aminotransferase 2-like 1	Agxt2l1	71760	−2.13
Sulfotransferase family 1A, phenol-preferring, member 1	Sult1a1	20887	−2.05
Solute carrier family 15 (H+/peptide transporter), member 2	Slc15a2	57738	−2.03
Connective tissue growth factor	Ctgf	14219	−1.99
DNA segment, Chr 14, ERATO Doi 449, expressed	D14Ertd449e	66039	−1.97
Contactin 4	Cntn4	269784	−1.96
White adipose tissue			
Predicted gene 10002	Gm10002	791405	−4.98
Killer cell lectin-like receptor subfamily B member 1B	Klrb1b	80782	−3.7
Predicted gene 2663	Gm2663	100040208	−3.23
Pyruvate dehydrogenase kinase, isoenzyme 4	Pdk4	27273	−2.88
Solute carrier family 15 (H+/peptide transporter), member 2	Slc15a2	57738	−2.75
Arginase type II	Arg2	11847	−2.64
CD5 antigen-like	Cd5l	11801	−2.48
Cysteinyl leukotriene receptor 2	Cysltr2	70086	−2.48
Transient receptor potential cation channel, subfamily V, member 6	Trpv6	64177	−2.47
3-hydroxy-3-methylglutaryl-Coenzyme A synthase 2	Hmgcs2	15360	−2.47
Gastrocnemius muscle			
Indolethylamine N-methyltransferase	Inmt	21743	−7.33
RIKEN cDNA 8430408G22 gene	8430408G22Rik	213393	−6.05
Chemokine (C-X-C motif) ligand 13	Cxcl13	55985	−5.57
ChaC, cation transport regulator 1	Chac1	69065	−5.51
CCAAT/enhancer binding protein (C/EBP), delta	Cebpd	12609	−4.85
F-box protein 32	Fbxo32	67731	−4.79
Arrestin domain containing 2	Arrdc2	70807	−4.40
Sestrin 1	Sesn1	140742	−4.31
Solute carrier family 43, member 1	Slc43a1	72401	−4.30
DNA-damage-inducible transcript 4	Ddit4	74747	−4.18

**Table 3:** Obesity-associated genes that show altered expression in FTO-4 mice.

Hypothalamus			
*Gene name*	*Gene Symbol*	*Entrez ID*	*Fold change*
Carbonic anhydrase 2	Car2	12349	1.59
Growth hormone	Gh	14599	−14.89
Pyruvate dehydrogenase kinase, isoenzyme 4	Pdk4	27273	−1.58
Cerebellum			
Delta-like 1 homolog (Drosophila)	Dlk1	13386	−1.55
Uncoupling protein 2 (mitochondrial, proton carrier)	Ucp2	7351	−1.50
White adipose tissue			
Adiponectin, C1Q and collagen domain containing	Adipoq	11450	3.26
Adrenergic receptor, beta 3	Adrb3	11556	2.59
Androgen receptor	Ar	11835	1.70
Carbonic anhydrase 3	Car3	12350	4.20
Caveolin 1, caveolae protein	Cav1	12389	1.89
Cytochrome P450, family 2, subfamily e, polypeptide 1	Cyp2e1	13106	3.83
Leptin	Lep	16846	2.72
Monoamine oxidase B	Maob	109731	1.68
Pyruvate dehydrogenase kinase, isoenzyme 4	Pdk4	27273	−2.88
Perilipin 1	Plin1	103968	2.64
Gastrocnemius muscle			
Adrenergic receptor, beta 2	Adrb2	11555	−1.78
CCAAT/enhancer binding protein (C/EBP), beta	Cebpb	12608	−1.96
Eukaryotic translation initiation factor 4E binding protein 1	Eif4ebp1	13685	−2.74
Heparin-binding EGF-like growth factor	Hbegf	15200	1.91
Interleukin 6 receptor, alpha	Il6ra	16194	−3.61
Insulin receptor substrate 1	Irs1	16367	1.70
Insulin receptor substrate 2	Irs2	384783	−2.10
Low density lipoprotein receptor	Ldlr	16835	1.66
Lipin 2	Lpin2	64898	−1.80
Neuropeptide Y receptor Y1	Npy1r	18166	−1.60
nuclear receptor subfamily 4, group A, member 3	Nr4a3	18124	2.01
Phosphodiesterase 4A, cAMP specific	Pde4a	18577	2.15
Phosphodiesterase 7A	Pde7a	18583	−1.98
Phosphodiesterase 7B	Pde7b	29863	1.72
Pyruvate dehydrogenase kinase, isoenzyme 4	Pdk4	27273	−1.56
Paternally expressed 3	Peg3	18616	−1.69
Peroxisome proliferative activated receptor, gamma, coactivator 1 beta	Ppargc1b	170826	2.10
Retinoid X receptor gamma	Rxrg	20183	2.09
Serine (or cysteine) peptidase inhibitor, clade E, member 1	Serpine1	18787	−1.62
Vascular endothelial growth factor A	Vegfa	22339	1.71

### Changes in gene expression common to several tissues

Several genes showed same-direction changes in expression in multiple tissues. For example, *Glo1* was upregulated in all four tissues examined. *Glo1* encodes an enzyme involved in the detoxification of methylglyoxal (a metabolic by-product of glycolysis) and glutathione, and has been linked to dietary obesity in various QTL studies in mice [28], [29]. *Wdfy1*, also known as *FENS-1*, showed markedly increased expression in hypothalamus, cerebellum and gastrocnemius muscle. *FENS-1* protein has been reported to localise to early endosomes within the cell [30] and it contains a phosphatidylinositol-phosphate (PI3)-binding FYVE domain potentially allowing it to participate in the PI3 kinase signal transduction cascade.

Nothing is known of the function of this gene but as it contains a phosphatidylinositol-phosphate (PI3)-binding FYVE domain it may participate in the PI3 kinase signal transduction cascade.

*Pyruvate dehydrogenase kinase 4 (PDK4*), which plays an important role in the regulation of glucose utilization and lipid metabolism [31], was downregulated in the hypothalamus, WAT and gastrocnemius muscle of FTO-4 mice. The expression of *xanthine dehydrogenase* (*XDH*), which is involved in the oxidative metabolism of purines, was reduced in hypothalamus, cerebellum (1.46 fold) and gastrocnemius muscle.

*D14Ertd449e* (human *C10orf57*), *Plac9* and *Duxbl* were all downregulated in both hypothalamus and cerebellum of FTO-4 mice. *Duxbl* was downregulated 1.42 fold and 1.35 fold in hypothalamus and cerebellum respectively. In addition, *D14Ertd449e* was downregulated in WAT and *Plac9* in gastrocnemius muscle.

### Changes in gene expression specific to certain tissues

Several genes associated with inhibition of growth and/or proliferation were downregulated in the hypothalamus of FTO-4 mice. This included the cell cycle inhibitors CDKN1A and CDKN1B, the protein synthesis inhibitor SGK1, and TXNIP (which is involved in unfolded protein response, and whose activation leads to apoptosis).

In WAT, major urinary protein (MUP) genes were highly upregulated in FTO-4 mice. MUPs have been implicated in many other physiological processes, including the regulation of glucose and lipid metabolism [32] and energy expenditure [33]. Adiponectin was upregulated in WAT and its receptor AdipoR2 was downregulated 1.38 fold in the hypothalamus of FTO-4 mice. Expression of the anorexigenic hormone leptin was also increased. Leptin is expressed in proportion to body fat content [34], and as FTO-4 mice increase their adipose tissue mass, leptin expression is likely to rise. CYP2E1, an enzyme that participates in drug metabolism and oxidation of several fatty acids, was also upregulated in adipose tissue. Its activity results in the production of reactive oxygen species and CYP2E1 KO mice are protected against diet induced obesity. [35] Furthermore, CYP2E1 protein levels are increased in rodents on a high-fat diet. [36]

The most striking feature relating to gastrocnemius muscle is that the fold-increases in gene expression were generally larger than those in other tissues. NMRK2, which is involved in myogenesis and is largely muscle-specific, was upregulated in FTO-4 mice. In addition, the transcriptional activator CITED4 and the mitochondrial translational activator ZMYND17 were upregulated. These changes are consistent with the small expansion of muscle mass observed in FTO-4 mice [9]. In addition, both the *persoxisome proliferator-activated receptor gamma coactivator-1B* (PPARGC1B) and the *retinoid X receptor* RXRG were upregulated. PPARGC1B is a transcriptional co-activator that coordinates metabolic gene programs in response to environmental cues, and in muscle it stimulates mitochondrial biogenesis. The PPARGC1-family acts as a co-activator for the transcription of retinoid X receptors (RXRs). [37]

### Pathway analysis

To examine pathways that might contribute to the phenotype observed in FTO-4 mice, we carried out pathway analysis using GO-Elite. Pathways in which more than 20% of the genes showed changes in expression in FTO-4 mice are listed in Tables S3 (GO-Name) and S4 (Map Annotator and Pathway Profiler (MAPP)) in File S1.

Pathway analysis showed that anabolic pathways in WAT and skeletal muscle were significantly associated with FTO-4 mice, which is in line with the increased body weight of these mice. Furthermore, analysis revealed that a general methylation pathway contained a high percentage of genes (4 out of a total of 9 genes) that showed altered expression in gastrocnemius muscle (Table S4 in File S1). These included *Tpmt* (thiopurine S-methyltransferase) and *Mat2a* (methionine adenosyltransferase II alpha), which were upregulated, and *Inmt* (indolethylamine N-methyltransferase) and *Pnmt* (phenylethanolamine N-methyltransferase), which were downregulated.

### Influence of FTO on m6A levels

**Figure 3:**
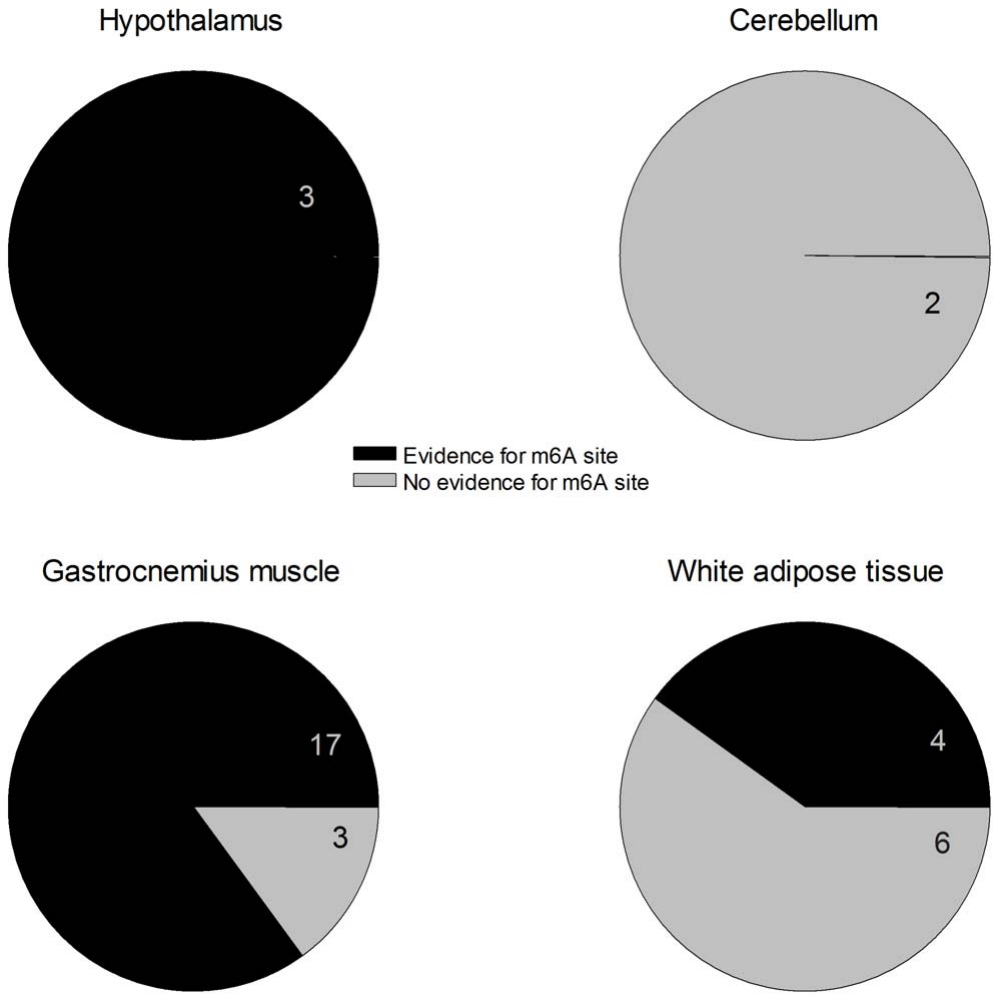
Effect of FTO overexpression on m6A levels in RNA. FTO mRNA expression in wild-type (black bar) and FTO-4 (white bar) MEFs as the fold change compared to wild-type (A). m6A levels measured as a percentage of adenosine levels for mRNA and total RNA by LC/MS in wild-type (black bar) and FTO-4 (white bar) MEFs (B). Significance was tested using Student's t-tests to compare FTO-4 to WT data. **p<0.01.

Because genes involved in methylation showed altered expression levels in the gastrocnemius muscle of FTO-4 mice and FTO is a known demethylase of single stranded DNA and RNA, we investigated whether FTO affected m6A levels. Comparing MEFs from wild-type to FTO-4 mice, FTO was clearly upregulated in FTO-4 MEFs (Figure 3A). Subsequently, the ratio of m6A to A was determined in both total RNA and mRNA in wild-type and FTO-4 MEFs. This was accomplished by RNA extraction, enzymatic hydrolysis to free nucleotides using nuclease P1 and dephosphorylation to nucleosides by alkaline phosphatase. Concentrations of individual nucleosides were determined by LC-MS using HILIC chromatography with isocratic elution and positive mode electrospray mass spectrometry with ^13^C5-adenosine as an internal standard. From the obtained concentrations, ratios of m6A to A were calculated and used as a measure of m6A level for comparison between different samples. FTO overexpression was found to have no effect on m6A levels in both total RNA and mRNA (Figure 3B).

## Discussion

Beginning at around 5 weeks of age, FTO-4 mice exhibit increased body weight, increased fat mass and slightly increased lean mass.[9] It is therefore of interest that significant changes in expression of a number of genes associated with growth and/or proliferation were observed in the hypothalamus. Likewise, in gastrocnemius muscle, genes potentially involved in the proliferation of muscle mass were upregulated. Several genes whose expression was altered were also associated with metabolism and/or obesity. Furthermore, pathway analysis revealed the association of several anabolic pathways in skeletal muscle and WAT with FTO-4. Thus the changes in gene expression we observe may underlie the increase in body weight observed in FTO-4 mice that is initiated at around 5 weeks of age and becomes significant in later life.

### Changes in gene expression

*D14Ertd449e* (human *C10orf57*), *Plac9* and*Duxbl* were downregulated in several tissues. Interestingly, all three of these genes are located close to one another on mouse chromosome 14. In C57/BL6 mice, *D14Ertd449e*, *Plac9* and*Duxbl* exist as three tandem repeats; but in other mouse strains, the number of repeats varies [38]. In humans, *C10orf57* and *Plac9* are also located in close vicinity to one another on chromosome 10, although not as tandem repeats.

In WAT, gene expression of two important hormones was altered. Leptin mRNA was increased, in line with the increase in fat mass in FTO-4 mice. Interestingly, leptin was found to mediate FTO expression via STAT3 in the brain [39]as well as in the liver [40]. Moreover, overexpression of FTO in the liver reduced leptin-induced phosphorylation of STAT3, which impaired leptin-induced signaling pathways and resulted in downregulation of leptin-regulated genes.[40]. In addition, liver FTO overexpression in mice led to increased levels of leptin. [40] These data suggest that the increase in leptin mRNA might be influenced by both an increase in fat mass in FTO-4 mass, and by a link between FTO and leptin signalling.

Adiponectin was upregulated in WAT and its receptor AdipoR2 was downregulated 1.38 fold in the hypothalamus of FTO-4 mice. The hormone adiponectin is exclusively secreted from adipose tissue and has been implicated in several metabolic processes, such as improvement of insulin sensitivity and fatty acid oxidation. [41] In contrast to the increase in gene expression of adiponectin we observed at 5 weeks of age, 20 week-old male FTO-4 mice showed a decrease in plasma adiponectin levels. [9] This reduction in plasma adiponectin levels was also observed in rodents on a high fat diet [42], [43] and in humans with obesity and insulin resistance. [44]–[46] However, the reduction in adiponectin levels might take longer to occur, as the stimulatory effect of caloric restriction on adiponectin gene expression was only detectable after long-term caloric restriction and not after short-term restriction. [47]

In WAT, major urinary protein (MUP) genes were highly upregulated. MUPs are encoded by 20 discrete isoforms in mice, which show >85% identity in DNA sequences. They are members of the lipocalin family of proteins and are abundantly expressed in the liver. In urine, MUPS act as pheromones [48], [49]. They have also been implicated in many other physiological processes, such as the regulation of glucose and lipid metabolism [32] and energy expenditure [33]. MUP gene expression is likely to be regulated by metabolism as MUP genes are down-regulated in liver [50] and subcutaneous adipose tissue [51] in response to dietary restriction. On the other hand, MUP gene expression is also decreased in WAT [52] and liver [33] of obese mice and in the liver in response to refeeding [32].

In gastrocnemius muscle, several genes and pathways related to muscle differentiation and growth were differentially regulated in FTO-4 mice, which is in line with their small increase in muscle mass. In future studies, it would be interesting to examine the effect of FTO overexpression on proliferation and differentiation of myotubes.

Interestingly, pathway analysis revealed that a methylation pathway that includes methyl transferases and enzymes involved in the biosynthesis of S-adenosylmethionine was significantly represented in genes differentially expressed in gastrocnemius muscle of FTO-4 mice. Of the four genes with altered expression levels in this pathway, *Mat2a* is of most interest. It catalyzes the production of S-adenosylmethionine, which is the major biological methyl donor in cellular processes, including the formation of m6A. [53]–[55] The increased expression of *Mat2a* in FTO-4 mice might serve as a compensatory mechanism to counteract the potential demethylation by FTO.

### Comparison with other studies

Changes in gene expression in mice carrying a loss-of-function point mutation in the FTO gene have been reported [8]. Several of these genes showed a significant change in the opposite direction to those seen when FTO is overexpressed (this study). These included *D14Ertd449e, C6, Plk2, Fat3* and *Gm4841*. For example, *D14Ertd449e* was upregulated in hypothalamus and WAT of mice carrying the FTO mutation, but downregulated in the same tissues in FTO-4 mice. This gene encodes a transmembrane protein of unknown function. Similarly, expression of *C6, Plk2* and *Fat3* in WAT and *Gm4841* in gastrocnemius muscle was reduced in FTO mutant mice, but increased in FTO-4 mice. The role of these genes in body weight regulation remains to be explored. *C6* is part of the complement system, its enhanced expression potentially may relate to the increased inflammatory response in obesity. *Plk2* is a serine-threonine protein kinase required for centriole duplication, and may therefore be related to the observed expansion of lean mass in FTO-4 mice. Fat3 may be involved in cell adhesion, via its cadherin domains: the role of *Gm4841* is unknown.

Microarray studies have also examined changes in gene expression when FTO is overexpressed in tissue-cultured HEK293 cells [56] and in human myotubes [57]. In the HEK293 cells, only one gene showed a change in expression in the same direction as in our *in vivo* study. This was the *Cmbl*, a cysteine hydrolase, which was upregulated in both HEK293 cells and in gastrocnemius muscle. The differences between the two studies may relate to the fact they were carried out under very different conditions (48-hours post-transfection in tissue culture, versus our *in vivo* study). FTO overexpression in human myotubes resulted in an overrepresentation of mitochondrial genes that changed expression. Three of these genes were significantly upregulated in FTO overexpressing myotubes as well as in skeletal muscle of FTO-4, namely *Cyb5b*, *Ppif*, and *Taco1*. Interestingly, our study found an overrepresentation of genes involved in positive regulation of mitochondrial depolarization in gastrocnemius muscle of FTO-4 mice (Table S3 in File S1).

### Comparison with m6A studies

**Figure 4:**
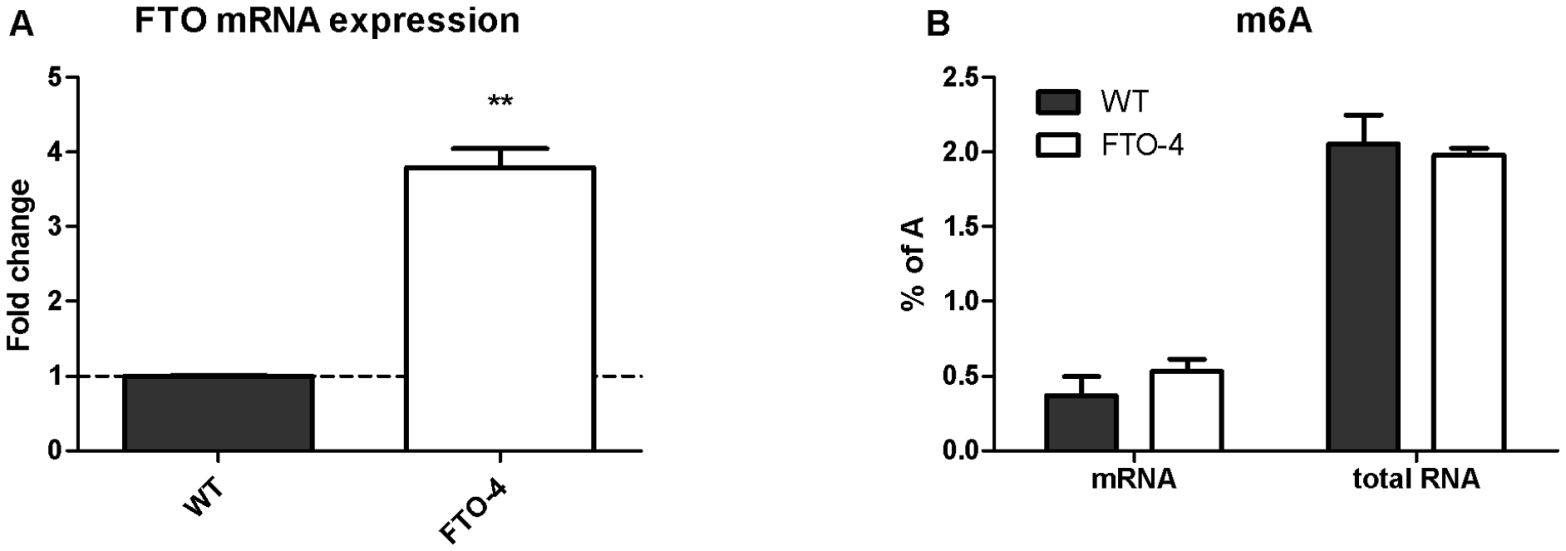
Overview of potential m6A sites in obesity-related genes with changed expression in FTO-4 mice. Pie chart of obesity-related genes whose expression is changed in the indicated tissues of FTO-4 mice. Dark grey, m6A sites in their mRNA transcripts [23], [24]. Light grey, no m6A sites [23], [24]. The number of genes involved is indicated: see Table 2 for gene names.

Two studies have identified m6A-containing mRNAs in mouse liver [24] and brain [23]. As FTO is known to demethylate mRNA at m6A [10], [12], we compared whether mRNAs that alter their expression in FTO-4 mice have been reported to contain m6A (Table S5 in File S1). Most FTO-regulated genes in gastrocnemius muscle that are known to be associated with obesity were found to contain m6A (Figure 4). In the hypothalamus, all FTO-regulated genes that are known to be associated with obesity were described to contain m6A sites. However, only 3 FTO-regulated genes have been associated with obesity in the hypothalamus, compared to 20 genes in gastrocnemius muscle. Although some genes that alter their expression in cerebellum and WAT of FTO-4 mice also contained m6A, the numbers were not as dramatic as in gastrocnemius muscle. Nevertheless, the data are compatible with the idea that FTO might influence the expression of at least some genes via regulating the amount of m6A in their mRNA.

To examine the effect of FTO overexpression on m6A levels, MEFs were isolated from wild-type and FTO-4 mice. Quantitative nucleoside analysis revealed that overexpression of FTO did not attenuate m6A levels in either total RNA or mRNA. Similarly, others have failed to see an effect of FTO knockdown on m6A levels in total RNA in mouse brain. [56]. A previous study reported a decrease in m6A levels in mRNA of HeLa cells in response to FTO overexpression and an increase in mRNA m6A levels following silencing of FTO in both HeLa and 293FT cells.[12] This study differed from ours in that FTO overexpression was higher (6 to 8-fold on protein level) and only acute effects of overexpression (24 hrs) were examined.[12] MEFs taken from FTO-4 embryos might have been adapted to high levels of the demethylase FTO, which could mask the effect of FTO on m6A levels. m6A methylation is a very dynamic process, and small differences in conditions could affect m6A levels. By using littermates for generating the MEFs, and also in the microarray study, we tried to avoid as much variation as possible. Our results do not provide evidence that under physiological conditions, overexpression of FTO lead to changes in total and mRNA m6A levels.

### Conclusion

This study examined the changes in gene expression in FTO-4 mice before the onset of obesity at 5 weeks of age. Our data revealed that anabolic pathways and genes are already activated at this time-point, consistent with the increased body weight and adiposity observed in these mice at an older age [9]. FTO is known to demethylate single-stranded DNA and RNA. Interestingly, a methylation pathway in skeletal muscle was associated with FTO overexpression. Moreover, the majority of FTO-regulated genes that were previously associated with obesity in this tissue are likely to contain m6A sites in their mRNA transcripts. Hence regulation of expression of these genes potentially could be mediated by FTO. However, we failed to detect an effect of FTO overexpression on general m6A levels in either mRNA or in total RNA derived from MEFs. This suggests any effect of FTO on m6A methylation is gene specific and involves only a small subset of mRNAs. This stresses the importance of investigating the direct mRNA targets of FTO, as has been done in the case of ghrelin [4] and genes involved in dopaminergic signalling. [25]
